# Validation of a Bayesian Adaptive Estimation Technique in the Stop-Signal Task

**DOI:** 10.1371/journal.pone.0165525

**Published:** 2016-11-23

**Authors:** Evan J. Livesey, David J. Livesey

**Affiliations:** School of Psychology, The University of Sydney, NSW, 2006, Australia; Brown University, UNITED STATES

## Abstract

The Stop Signal Task (SST), a commonly used measure of response inhibition, uses standard psychophysical methods to gain an estimate of the time needed to withhold a prepotent response. Under some circumstances, conventional forms of the SST are impractical to use because of the large number of trials necessary to gain a reliable estimate of the speed of inhibition. Here we applied to the SST an adaptive method for estimating psychometric parameters that can find reliable threshold estimates over a relatively small number of trials. The Ψ adaptive staircase, which uses a Bayesian algorithm to find the most likely parameters of a psychophysical function, was used to estimate the critical stop signal delay at which the probability of successful response inhibition equals 0.5. Using computational modeling and adult participants, estimates of stop signal reaction time (SSRT) based on the Ψ staircase were compared to estimates using the method of constant stimuli and a standard staircase method of adjustment. Results demonstrate that a reliable estimate of SSRT can be gained very quickly (20–30 stop trials), making the method very useful for testing populations that cannot maintain concentration for long periods or for rapidly obtaining multiple SSRT estimates from healthy adult participants.

## Introduction

Preventing oneself from performing an action is a fundamental part of normal response control and there are countless situations in which the most reflexive or pre-potent response must be inhibited in order to successfully achieve a goal or behave appropriately in a given context [1, 2]. Typically, the speed with which a participant is able to inhibit a pre-potent response is taken as a measure of response inhibition (RI). However, this can only be measured indirectly, since successful RI effectively eliminates an observable response. The stop-signal task (SST), as described by Logan & Cowan [1], has become a popular method for measuring the speed of RI. A typical SST comprises a large number of “go” trials on which participants are cued to give a prescribed response, and a smaller proportion of “stop” trials, on which the cue to respond (the go signal) is followed after a short interval by a stop signal instructing them to withhold that response. Response time (RT) on go trials and the probability of successfully inhibiting the response on stop trials are both critical for measuring RI in the SST. The interval between the go and stop signals, the stop-signal delay (SSD), is manipulated to find the critical delay at which the probability of successfully inhibiting the response equals 0.5. The difference between the average RT on go trials and this critical SSD reflects the average time that a participant takes to successfully withhold the response (stop-signal reaction time or SSRT). SSRT has proven to be a useful measure of individual differences in RI, Sergeant [3] describing the SST as ‘the most direct measure of the processes required in inhibiting a response’ (p9) and consequently it is now a widely used research and assessment tool, particularly in relation to developmental disorders such as Attention Deficit Hyperactivity Disorder (ADHD).

A number of studies, primarily using school-aged children and adolescents, have shown a link between ADHD and poor RI as measured by the SST and recent meta-analyses of such studies have identified SST performance as the most consistent difference between ADHD and control groups [4, 5, 6, 7]. These studies have focused on school-aged children and adolescent participants (plus a small number of studies with adults), thus little is known of the SST performance of younger children (with or without ADHD). The SST is problematic when testing younger children because of the large number of trials (on an uninteresting task) required to yield a stable estimate of SSRT. Although several comprehensive lifespan development studies have successfully measured response inhibition in children as young as six years [8, 9], gaining reliable measures of SSRT from younger children (especially children in the age range of 4–7 years) still remains a significant challenge for developmental researchers. Calculating SSRT requires manipulation of the SSD across stop trials. Traditional methods of adjusting the SSD mean that the task is relatively time-consuming as it takes many stop trials to reach stable estimates. To compound the problem, stop trials necessarily make up a small proportion of the total trials (around 25% in most cases) in order to maintain the pre-potency of the go responses. Therefore, there is a need for a version of the SST that is simpler, more engaging and also much shorter than conventional measures, so that it is appropriate for a wide range of ages, including preschool aged children. A ‘child-friendly’ version of the SST that was developed to be more visually stimulating and intuitive for young children revealed a developmental trend of improved performance across the age-range 4- to 7-years [10, 11, 12, 13]. However, the task still required many trials for reliable estimation of SSRT, thus taxing the attention span of the younger children (and of older participants with attention problems such as those found in ADHD). The aim of this study was to develop a means of estimating SSRT over a substantially reduced number of trials, and to begin to validate this version of the SST as a reliable measure of RI. Here we describe a technique that could be useful in gaining rapid estimates of SSRT and test its performance using monte-carlo simulations and normal adult participants.

Two conventional methods of presenting the go and stop trials have been widely used in the SST. The original SST used a method of constant stimuli, where stop signals are presented at set intervals after the go signal and an inhibition function is produced based on the probability of inhibition at each stop-signal interval [14]. The other is a nonparametric adaptive staircase which involves a simple method of adjustment whereby the SSD is increased or decreased after each stop trial in a stepwise fashion according to the success or failure to inhibit on the previous stop trial [15]. This homes in on the point at which the probability of inhibiting is 0.5, the point at which the best estimate of SSRT is obtained. Both of these methods require large numbers of trials, although adaptive stepwise adjustment is relatively rapid in comparison to the method of constant stimuli and hence has been the preferred method.

### Rapid threshold estimation using the Bayesian adaptive estimates technique

In psychophysics, a similar problem is well known to researchers requiring reliable estimates of psychophysical thresholds in situations where running large numbers of trials is impractical. Researchers have used Bayes’ theorem to develop several techniques to try to overcome the practical limitations of lengthy psychophysical procedures, thereby expanding the repertoire of analytic tools available for rapid threshold estimation [16, 17, 18]. Kontsevich and Tyler [18] outlined an adaptive estimation technique (the Ψ method) that takes advantage of Bayes’ rule and chooses test variables on the principle of minimizing entropy, the amount of information required to have complete knowledge of a system [19, 20], to find reliable threshold estimates over a relatively small number of trials. The technique calculates the prior probabilities of a successful response for each of several possible stimulus values that could be chosen to present to the observer, assuming that those probabilities are governed by an underlying psychometric function with a range of different possible parameters. The goal is to find the most likely combination of parameters (i.e. the best fitting psychometric function) within a defined parameter space. On each trial, the amount of information that could be gained from testing each stimulus value is calculated, and the stimulus value that stands to yield the most information is selected for the next trial. On the basis of the observer’s response, the posterior probabilities of each combination of parameters are then updated, ready to calculate entropy for selecting the next stimulus value. In this fashion, the Ψ staircase quickly finds a stable estimate of the most likely psychometric parameters. In our application to the SST, the successful response is actual inhibition of a response on stop trials, and the parameters being estimated describe the slope and threshold of the function that relates probability of successfully inhibiting, p(i), to the SSD. Thus the aim of the technique is to use the Ψ method to quickly and accurately estimate the critical SSD for which RI success and failure are equally likely.

This study compared and validated the Ψ method of interval adjustment against methods previously used with adults and children. The stepwise adjustment and Ψ methods were compared with functions derived from trials using the method of constant stimuli, a lengthier test procedure that is not practicable in many testing situations, but is nevertheless useful for further validation of the shorter estimation procedures.

The use of an adaptive estimation method may permit reliable estimation of the SSRT over a relatively small number of trials. Kontsevich and Tyler [18] demonstrated that reliable threshold estimates could be calculated with the Ψ method using as few as 30 trials on tasks that estimate psychophysical thresholds. However, this method was designed for and validated using two-alternative forced-choice psychophysical judgments made under carefully controlled conditions. It remains to be seen whether the same method will be useful under the response requirements of the stop signal task.

### Applying the Ψ method to the SST

We first tested the feasibility of using the Ψ staircase to estimate SSRT using simulations of response inhibition based on a simple horse-race model of RI [21]. The simulations followed a similar rationale and method to that used by Band, van der Molen, and Logan [22]. Go RTs were assumed to be distributed according to the concatenation of Gaussian and exponential distributions (an ex-Gaussian distribution). The ex-Gaussian distribution mimics the positive skew of most RT distributions from choice response tasks and several researchers have found that it yields a good fit to empirical data [23, 24, 25, 26]. This distribution is assumed to be the same for go and stop trials. In other words, the presence of the stop signal on a stop trial is assumed to have no impact on the speed of executing the go response, if indeed it is executed. Under a simple race model of response execution, on each stop trial, if execution of the inhibitory process finishes before execution of the primary response then the response is successfully withheld. If execution of the primary response finishes before execution of the inhibitory process then the response is made (a failure to inhibit).

A more comprehensive and detailed description of the Ψ method can be found in Kontsevich and Tyler [18]. We will focus here on the particular details of its implementation in the current paradigm. To begin with, the probability of successfully inhibiting a response on a stop trial must be assumed to be a function of the SSD. This is effectively a survival function, where the probability of successfully inhibiting decreases monotonically with the duration of the SSD. Alternatively, the function can be described just as validly as a cumulative function of the time remaining before the designated trial timeout at the onset of the stop signal (~SSD). As the SSD approaches the time when the trial times out (~SSD = 0), the probability of successful inhibition should be very low, equal to a baseline error rate reflecting the proportion of go trials on which a go response is not performed in the allotted time. When SSD equals zero (~SSD equals the maximum time allowed to make the response), the probability of successful inhibition should be very high, equal to one minus the proportion of trials on which the stop signal is completely ignored regardless of the SSD. The following equations provide two methods for deriving the probability of successful inhibition, p(i) as a function of the time remaining between the SSD and the end of the trial, ~SSD:

Eq 1: Normal cumulative distribution function (CDF)

$$\text{P}\left(\text{i}\right)=\left(1-2\text{E}\right)\times \frac{1}{2}\left(1+\text{erf}\left(\frac{\sim \text{SSD}-\;\alpha }{\beta \;\times \;\sqrt{2}}\right)\right)+\text{ E}$$ (1)

Eq 2: Weibull CDF with exponent of e

$$\text{P}\left(\text{i}\right)=\left(1-2\text{E}\right)\times \left(1-{\text{e}}^{-{\left(\frac{\sim \text{SSD}}{\alpha }\right)}^{\beta }}\right)+\text{ E}$$ (2)

In the normal CDF (Eq 1), α is the mean of the normal function and also the point where p(i|~SSD) = 0.5 (the critical ~SSD), whereas β is the standard deviation and affects the slope of the function. In the Weibull functions, α is the scale parameter and β the shape parameter. E refers to the error rate where the participant fails to inhibit their response regardless of the SSD, but also to the rate of forced inhibition, where the individual makes no attempt to perform (or at least complete) the go response, regardless of the SSD. In practice, and particularly when using the SST with children, these two sources of error often prevent perfect inhibition at very short SSDs and perfect failure of inhibition at very long SSDs, respectively. Although there is no reason to assume they will be equal, for the sake of simplicity we implement their influence on the p(i) function with a single fixed parameter E. Note that although errors of this sort are rare when testing healthy adult populations, it is essential to include this error rate because otherwise a single error at an easy SSD or single correct inhibition at a very difficult SSD can have a disproportionately strong effect on the estimate. We have generally opted to set E = .04, which may well be overestimate for healthy adults but realistic for work with young children or other populations where basic task compliance can be intermittently interrupted. Unlike the normal CDF, the Weibull functions are not symmetrical around p(i ~SSD) = 0.5 and therefore could be argued to describe the predicted function for a skewed RT distribution more accurately. For convenience, we suggest using Eq 3, which differs from Eq 2 only in that it uses a base of 2 rather than a base of e. Like a normal CDF, a Weibull CDF with a base of 2 conveniently uses a parameter (α) which is equivalent to the critical ~SSD, whereas the same parameter in Eq 2 corresponds to approximately p(i|~SSD) = 0.62, meaning the critical ~SSD needs to be calculated using the best estimates of both the shape and scale parameters.

Eq 3: Weibull CDF with exponent of 2

$$\text{P}\left(\text{i}\right)=\left(1-2\text{E}\right)\times \left(1-2^{-{\left(\frac{\sim \text{SSD}}{\alpha }\right)}^{\beta }}\right)+\text{ E}$$ (3)

We chose to consider normal and Weibull CDFs because both have been used successfully with the Ψ in past applications aimed at finding psychophysical thresholds (e.g. [18, 27]), in situations where the underlying function is assumed to be sigmoidal. It should be noted, however, that they are not commonly used in the SSRT literature and other functions may well produce fits to p(i) data that are as good or better e.g., see Logan & Cowan [1] for an alternative approach]. We considered the Weibull CDF in addition to the normal CDF because, as noted above, the Weibull is not symmetrical around the threshold level indicated by α, and thus it potentially could be more useful for modeling stopping data derived from skewed distributions. However, in practice, we have found that both of these functions fit stopping data well and with approximately equal precision.

Using the Ψ staircase, a sequence of steps is iterated on each stop trial to update the best parameter estimate and select the value to test on the next trial. The equations governing these steps are detailed formally by Kontsevich & Tyler [18] but will be described briefly here in terms of the current paradigm. Using either a normal or Weibull function, the technique requires consideration of the likelihood of various values taken by the α and β parameters, and the probability of events given each combination of α and β values. To implement this, a parameter space is first defined; within reasonable lower and upper limits, monotonic increments in each parameter are considered. Whereas it is often advantageous to consider steps in log units when estimating intensity-based psychophysical thresholds, for estimating p(i) as a function of delay, we considered equidistant steps in milliseconds. Whereas selection of the upper and lower bounds of the parameter space can be critical (as discussed and illustrated later), the choice of the number of steps between bounds may ultimately be determined by pragmatic limitations such as processing speed. For instance, we have found that using a parameter space with steps in α of between 1 and 20ms produce similar results, but the more fine-grained the steps, the longer the calculations will take. The resulting two-dimensional parameter space forms the basis of likelihood estimates for the observed data, and for estimated probability of successful inhibition given each combination of SSD and parameters α and β.

The first iterative step involves calculating the probability of each response outcome (successful inhibition or failure to inhibit the go response) for each possible SSD that could be chosen on the next trial. This calculation involves computing the probability of each of the two outcomes, for each combination of SSD, α and β parameters, then weighting those probabilities according to the prior probability of each combination of α and β parameters. At first, these prior probabilities are uniform as each set of parameters are considered equally likely, but with each stop trial, they are updated according to the probability of the observed outcome at the selected SSD.

An important innovation of the Ψ method is the manner in which the selection of the SSD occurs. *Before* selecting which SSD to use for the upcoming stop trial, Bayes’ rule is used to estimate the posterior probability of the α and β parameters given each possible set of events that might occur on the next trial. Hence the posterior is calculated for each combination of a) SSD that could be selected to present on the next trial, and b) potential response outcome, either a successful inhibition or an unsuccessful go response. For each of these probability density functions, the entropy can then be estimated, yielding a measure of the uncertainty that would be left if that SSD was presented and was met with that particular response outcome. The key value that the algorithm estimates is the expected entropy for each candidate SSD; whichever SSD is likely to yield the greatest reduction in entropy is the most useful to test next.

To find the test value with the greatest potential to reduce uncertainty, the expected entropy for a given SSD can be estimated as a sum of the entropies of each possible response, weighted according to the probability that the response will occur. By definition, the maximum *reduction* in entropy occurs at a test value for which the outcome is likely to be relatively informative. In other words, according to the algorithm, the SSD with the minimum expected entropy is the most useful to test on the next trial. In practice, this value rarely resides at an SSD for which inhibition is either very likely or very unlikely; for these SSDs, the observed outcome is probably not going to yield much additional information about the true parameters. However, according to the algorithm, the critical SSD where the two responses are equally likely is generally not the most useful value to test either. Instead minimum expected entropy often corresponds to an SSD that is moderately longer or shorter than the critical SSD.

Once these estimates of entropy have been computed, the SSD with the minimum expected entropy is selected to present on the next stop trial. This selected value is expected to yield the greatest increase in confidence about the true underlying parameters. The stop trial is then run, and the participant either successfully inhibits or fails to do so. The appropriate posterior probability distribution, which has already been calculated using Bayes’ theorem to select the SSD, can then be chosen depending on which response was made. The best estimate of the psychometric function is calculated by taking the mean of this distribution. The mean is generally regarded to yield a more reliable estimate of the underlying parameters than simply taking the (α, β) coordinates with the maximum probability [18, 28].

## Simulations of SSRT Estimation

The major potential advantage of using the Ψ staircase is the speed with which it achieves a reliable estimate of the critical SSD. Thus it is important that variations in Ψ estimates reflect real variations in the underlying speed of inhibition and also that the staircase is potentially sensitive enough to measure small differences in SSRT at the group level. We tested these by running a series of simulations using parameters that reflect typical estimates for Go RT and a range of SSRTs that span those likely to be observed in healthy adults. To begin with, we ran 25 simulated experiments in which, for each simulation, we systematically varied stop RT in fixed steps ranging from 52 to 248 ms, spaced 4 ms apart. In these simulations, the go RTs were always sampled from an ex-Gaussian distribution with μ = 390ms, δ = 40ms, τ = 60ms, though we have observed similar results with a range of other go distribution parameters. These parameters were used to create a distribution of 10000 responses with an average sample mean of 450ms and average SD of 72ms. In essence, these simulations emulate a situation in which we have a sample of 50 participants with equally spaced inhibition speeds but the same go response characteristics. Although clearly very artificial, these simulations provide a basic first test of the properties of the staircase.

For each simulated participant, one hundred go trial RTs were randomly selected for each SSD estimate and used as the would-be go response speeds for 100 stop trials. Whether response inhibition was deemed to be successful or unsuccessful was then determined by a comparison between the go RT and the sum of the stop RT and the SSD selected for that trial. Following the assumptions of a simple horse-race model, the process that finishes execution first dictates the binary outcome of that trial. Hence, response inhibition is successful if Go RT > (SSD + Stop RT), and response inhibition is unsuccessful if Go RT < = (SSD + Stop RT). We used a constant SSRT for these simulations rather than a SSRT distribution. It should be noted that this is a simplification of the likely mechanisms underlying response inhibition and differs from some recent studies that focus on the distribution of stopping times in SST. For instance, Matzke, Dolan, Logan, Brown & Wagenmakers [29] modeled stopping times using an ex-Gaussian distribution of the same form as the observable go RT distributions. Importantly, the relative strengths and weaknesses of the staircases discussed and illustrated below do not change substantially if one uses an SSRT distribution rather than a constant SSRT.

**Fig 1:**
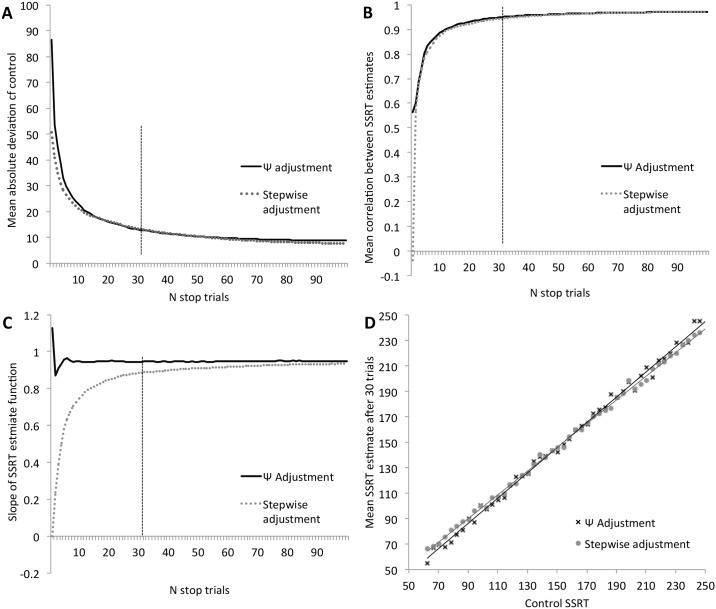
Results from horse-race model simulations with fixed Go RT distribution and varied stop RT. Stop RT was systematically varied around the starting point of the stepwise-adjusted staircase and across the parameter space considered by the Bayesian Ψ staircase. (A) Mean absolute deviation from the control SSRT as a function of staircase stop trial. (B) Mean correlation between SSRT estimates as a function of staircase stop trials. (C) Slope of the best fitting affine function describing the relationship between actual SSRT and estimated SSRT (a slope of less than 1 suggests overestimation of fast SSRT and underestimation of slow SSRT). (D) Mean SSRT estimate after 30 stop trials plotted against control SSRT.

We were interested in two aspects of the results from these simulations. First, how quickly and reliably the adaptive staircase method settled on an accurate estimate of SSRT relative to a typical stepwise-adjusted staircase, second whether the estimates were accurate or showed evidence of systematic bias across a range of SSRTs. To examine how quickly the staircases could find an accurate SSRT estimate, we calculated a control estimate of SSRT using the whole population of Go RTs in the distribution, then calculated the mean absolute difference between this control SSRT and the estimate after each trial of the staircase. For this first set of simulations, the stepwise-adjusted staircase always began with SSD of 300 ms, while the Ψ staircase considered a range of values for α that corresponded to an SSD between 50 and 550 ms. Fig 1A shows this mean absolute deviation from the control SSRT as a function of staircase stop trial, averaged across the simulations and across the different stop RTs. As is evident, both the method of stepwise adjustment and the Ψ method converge quickly on an accurate SSRT estimate. Both methods reached a mean deviation of less than 20 ms in less than 20 trials, and within 30 trials the mean deviation was less than 15 ms for both methods. Between 30 and 100 trials, the mean deviation improved marginally from 12 ms to 10 ms. For each simulated experiment, we calculated the correlation between the SSRT estimate from each staircase with the control SSRT (i.e. the correlation between measures amongst 50 simulated participants with systematically varied stop RT). These correlations were highly consistent across simulations; the means across the simulations are shown in Fig 1B. This shows that after 20 trials the correlation was approximately 0.92 and by 30 trials this had risen to 0.95, with marginal increase beyond 30 trials.

To assess whether the estimates were accurate across the full range of stop RTs, we examined the function relating actual SSRT to estimated SSRT for each simulation (note that each simulation contained 50 “participants” with different stop RTs). For each simulation, we calculated the best fitting affine function. If the staircase is unbiased (if SSRT estimates are equally accurate across the range of stop RTs) then the slope of this function should be close to 1. Fig 1C shows that the Ψ staircase yields a relatively unbiased estimate after as few as 10 trials whereas the method of stepwise adjustment takes longer to achieve a relatively unbiased estimate. Note that in terms of mean deviation (Fig 1A), the stepwise-adjusted and Ψ staircases appear to perform equally well, and yet Fig 1C suggests that there is a stronger bias in estimates for the stepwise-adjusted staircase, particularly after relatively few stop trials. The reason for this discrepancy, and the bias present under the stepwise adjustment method, will become clearer in the next set of simulations. Essentially, estimates using the Ψ staircase change very quickly in the first few trials. Although the estimate is relatively noisy in these early trials, the error is not systematic (for instance, the size of the deviation is not related to whether the parameter space is centered around a value that is shorter or longer than the critical SSD). In contrast, the stepwise-adjusted staircase produces a gradually changing estimate, thus its estimate is related to whether the starting SSD is longer or shorter than the critical SSD, which is particularly pronounced early in the session when there have been relatively few stop trials but diminishes with increasing numbers of trials. Whereas the Ψ staircase very quickly approximates the most likely value for the SSD, the stepwise-adjusted staircase shifts towards its ultimate estimate relatively gradually. Fig 1D shows the mean SSRT estimate after 30 stop trials plotted against control SSRT for both staircase methods. This shows that after 30 stop trials, the stepwise-adjusted staircase still slightly overestimates very fast SSRTs and slightly underestimates very slow SSRTs while the Ψ method provides a more accurate estimate across the range.

Although the Ψ method reaches an estimate very rapidly, it is bounded by the hypothesis space that is defined in terms of the range of parameters that are considered. This is important, especially if testing a population that potentially has very widely varying capacity for response inhibition. The parameter α tracks ~SSD in our equations and is thus the crucial parameter for estimating the critical SSD. In setting up a hypothesis space for the possible values that α can take, it is important to consider all reasonably plausible values of the critical SSD and make sure they can be captured by values of α because the likelihood estimation technique will never return a value outside of the parameter space that it is considering (at the limit, it will return a value at the upper or lower boundary of the parameter space). The following simulations illustrate this limitation, as well as the limitations of the stepwise-adjusted staircase in terms of the speed with which it reaches an accurate estimate.

As with any method using Bayesian inference, defining an appropriate hypothesis space is a crucial component for implementing the Ψ staircase. We examined whether biases in the SSRT estimate were evident when the range of the hypothesis space was centered on a value that was faster than, slower than, or equal to the ~SSD expected from the true stop RT. We also illustrate how the estimate is affected when the true stop RT produces an expected ~SSD that is outside of the range that is considered in the hypothesis space. In each case, the stop RT was set at 150ms. With a mean Go RT of 450ms, the expected critical SSD will be around 300ms. Two ranges were used for the α parameter of the p(i) function. The first was 500ms, which might be considered an appropriate range for a normal population of adults. The second was a narrower range of 200ms. For each of these SSD ranges, simulations were run with the SSD range centered on 150, 200, 250, 300, 350, 400, and 450ms. Importantly, this means that for the wider α range staircase, the critical SSD always fell within the bounds of the parameter space, whereas for the narrower range staircase, the SSD ranges centered on 150 ms and 450 ms did not include the critical SSD and for the SSD ranges centered on 200 ms and 400 ms, the critical SSD coincided with one of the boundaries. Twenty-five simulated experiments were run for each combination of parameter midpoint (150 to 450 ms in 7 steps) and width (200 ms vs 500 ms).

**Fig 2:**
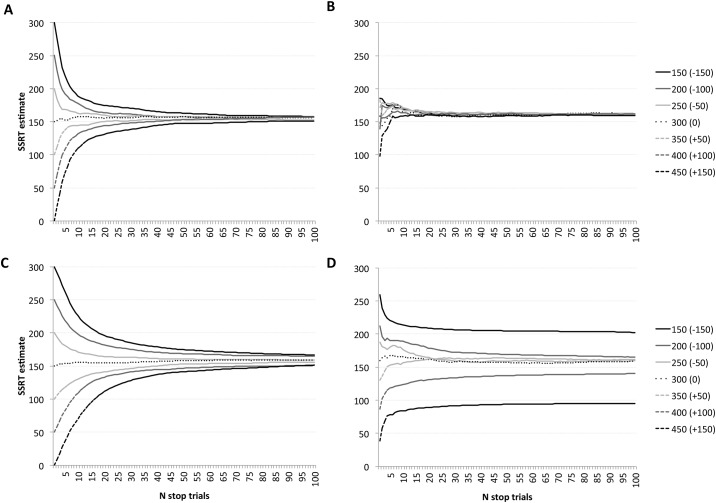
Mean simulated SSRT estimates across 100 stop trials. Simulations used adaptive staircases controlled by stepwise adjustment (A and C) or the Ψ method (B and D), in which the true stop RT was fixed at 150 ms. For stepwise adjustment, the numbers in the legend refer to the starting point for the SSD estimate (and the difference between the starting point and the actual difference between go RT and stop RT). The steps taken on each trial were either 50 ms (A) or 10 ms (C). For Ψ adjustment, the numbers in the legend refer to the mid-point of the hypothesis space for the alpha parameter (and the difference between this midpoint and the actual difference between go RT and stop RT). The range of the hypothesis space alpha parameter of the p(i) function was set at either 500ms (B) so that all critical SSDs fell within the hypothesis space or 200ms (D) so that the more extreme critical SSDs fell outside the hypothesis space.

These simulations were again compared with the stepwise-adjusted staircase used in the previous simulations. For the stepwise-adjusted staircase, we similarly examined how SSD estimates related to the starting value of the staircase. We used the same 7 values of the midpoints for the Ψ staircase parameter spaces as starting points, and adjusted the SSD on each trial in steps of either 10 ms (slow change) versus 50 ms (fast change). Since the SSD converges incrementally towards the critical SSD using stepwise adjustment, we would expect faster convergence with the larger step, but we anticipated that bias in SSD estimate discussed above in relation to the first simulations would be evident for both speeds, at least in the initial stop trials. Each simulation used an ex-Gaussian distribution of go RTs (μ = 390ms, δ = 40ms, τ = 60ms) and, as noted, a hypothetical stop speed of 150 ms. Results of these simulations are shown in Fig 2, with each data point representing the average across 25 simulations. This shows that when there is an appropriate range in the hypothesis space, the Ψ method (B) produces a reliable estimate of SSRT in many fewer trials than the stepwise adjustment method (A & C). However, when the range of the hypothesis space is narrow and the true stop RT produces an expected ~SSD that is outside of the range that is considered in the hypothesis space then the Ψ method produces estimates of the SSRT either above or below the true value (D). It is clear that when using the Ψ method the hypothesis space must have a wide range (well above and below the expected SSRT).

To complement the results of the simulations, two experiments were conducted to compare three methods of estimating the critical SSD, the method of constant stimuli, staircase using stepwise adjustment, and staircase use the Ψ method of adjustment.

## Experiment 1

In Experiment 1, a simple two-choice reaction time task was employed to compare the three methods of estimating the critical SSD. On each trial, a colored arrow appeared in the centre of the screen, pointing left or right, and the participant was required to respond as quickly as possible with a left or right key press. Stop trials constituted 25% of the total, and comprised intermixed trials on which the SSD was determined using the method of constant stimuli (CS stop trials), stepwise adjustment (SA stop trials), or the Ψ adaptive staircase (Ψ stop trials). Furthermore, two Ψ staircases were used concurrently, each using a slightly different sigmoid function for estimating probability of inhibition. Correlations between the estimates of the critical SSD gleaned from each method give an indication of the reliability of the new methods. SSRT was calculated using the difference between Go RT and the critical SSD, and this measure also provides a gauge of reliability of the new methods.

### Method

#### Participants and apparatus

Fifty-seven 1^st^ year psychology students from the University of Sydney participated in the experiment, each tested individually in a quiet room. In this task, we used the SSD estimate derived from the method of constant stimuli as a baseline with which to compare the staircase estimates. Therefore it is important that all participants produce a reliable estimate using the method of constant stimuli. For this reason, we excluded participants who produced a poor fit (r^2^ < .85) to Eqs 1–3 on the method of constant stimuli. Seven participants were excluded on this basis, leaving a sample of 50 (41 female) of mean age 19.78 years (SD = 3.35, range: 18–36 years). Participants were tested in small groups of up to four at a time. The experiment was run on Dell Optiplex computers with 17inch monitors, using software designed using Visual Basic. All responses were recorded using a standard computer keyboard and participants wore standard audio headphones.

#### Procedure

On arrival, participants were told that they were participating in an experiment looking at response control. They were told that on each trial, an arrow would appear in the middle of the screen, pointing either left or right, and that they should press the corresponding key as quickly as possible without sacrificing accuracy. They were then given a short block of 12 practice trials on the go task. After completing the go practice block, participants were further instructed that on some trials, a signal would appear indicating that they should withhold their normal response. This signal was the screen turning red and a loud beep sound delivered through headphones. Participants were given another practice block with 24 trials (6 of which were stop trials). Finally, before beginning the experimental trials, participants were told that the time between the arrow appearing and the stop signal appearing would vary and that it would not always be possible to withhold their response. They were told to make sure they did not slow down on the go task in order to successfully withhold their responses on stop trials. To help them monitor their response speed, average RT was displayed at the end of each block of 72 trials, along with the average RT for the previous block and the average RT for the first block.

Trials were organized in 20 blocks of 72 trials. Each block contained 54 go trials and 18 stop trials. Of the stop trials, 12 were CS trials, 2 were SA trials, 2 were Ψ (normal) trials where the probability of inhibition was calculated using a normal CDF, and 2 were Ψ (Weibull) trials where the probability of inhibition was calculated using a Weibull CDF function. In each block, each type of stop trial was assigned an equal number of left and right go cues. The go trials in each block also had an equal number of left and right go cues. Trial order within each block was randomized. The SA procedure employed adjustments of 50 msec.

#### Ethical statement

This study (and Experiment 2) was conducted with approval from the University of Sydney Human Research Ethics Committee. All participants were fully informed about the study and written informed consent was required for participation.

### Results

#### Estimates of the critical Stop Signal Delay

The mean critical SSD calculated using the method of constant stimuli was 141.42 ms (SD = 30.54 ms). For the two Ψ staircases the mean estimates after 40 trials were 136.36 ms (SD = 33.18 ms) and 138.62 ms (SD = 32.95 ms). The mean estimate after 40 trials of the stepwise-adjusted staircase was 127.82 ms (SD = 31.20 ms). Pairwise comparisons of these estimates, correcting for family-wise error rate, suggested that the stepwise adjustment yielded a significantly smaller estimate than both Ψ methods and the method of constant stimuli, smallest t(49) = 3.67, p < .05, but that the other three estimates did not significantly differ, largest t(49) = 2.13, p>.05.

**Fig 3:**
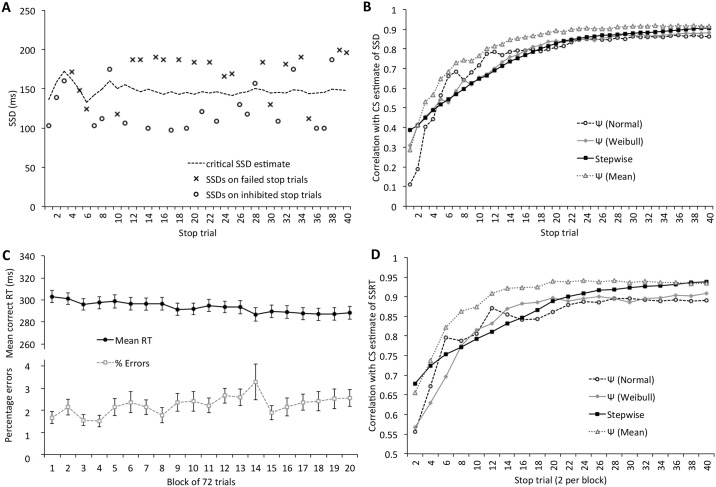
Estimates of critical SSD and response speed in Experiment 1. A) SSD estimate and values tested for a typical participant. B) Correlation between the (terminal) control CS estimate of SSD and each of the other three measures as a function of the number of stop trials completed. (C) Mean RT on correct go trials and % errors across the 20 blocks of trials. (D) Correlations between the CS estimate of SSRT and each of the other methods across trials.

Panel A of Fig 3 shows a typical example of the Ψ method estimate and the SSD test values chosen to maximise the information gained from each trial. As Kontsevich and Tyler [18] explained in detail in their original paper, the method tends to choose values above and below the current most likely estimate rather than values close to the estimate itself. However, as can be seen in Fig 3A, those values do not necessarily follow an obviously systematic or regular progression in the way that a stepwise-adjusted staircase would. Panel B of Fig 3 depicts the correlation between the SSD calculated using the method of constant stimuli and each of the staircases after 1–40 trials. All three staircases had correlations with the CS estimate greater than 0.80 after 20 trials and greater than 0.86 after 30 trials. Fig 3B also includes correlations between the CS estimate and the mean of the two Ψ estimates.

#### Go responses

Accuracy on the primary task was very high (mean = 97.76%, SD = 1.76%) and mean RT over all 20 experimental blocks was 293.38 ms (SD = 37.32ms). Fig 3C shows the progression of response time and errors across the experimental blocks. Since it is easier to successfully inhibit a response on a stop trial if one deliberately slows on the go response, it is important to determine whether RT increases across blocks. But in this case, the data suggest a very slight speeding of RT across blocks. Over the experimental blocks, RT decreased on average by 0.76 ms per block of 54 go trials.

#### SSRT estimates

Further to the estimation of SSD, estimates of SSRT are affected by changes in go RT estimation across the course of the procedure. Correlations between SSD estimates differ from correlations between SSRT estimates because the former are dependent on go RT, whereas the latter should at least in principle be fairly independent. Therefore, it is important to also examine how well progressive estimates of SSRT match with the control CS measure. SSRT is usually estimated by taking the difference between Go RT and critical SSD estimate. Thus, at the end of the experimental blocks, the SSRT estimates were 152.04 ms (SD = 32.85 ms) for the method of constant stimuli, 157.10 ms (SD = 36.95 ms) and 154.84 ms (SD = 37.09 ms) for the two Ψ staircases and 165.64 ms (SD = 37.69 ms) for the stepwise adjusted staircase. Note that since the Go RT estimate was the same for all SSRT calculations, wherever the SSD differed significantly between methods of estimation, so too does the SSRT estimate. As with the SSD estimates, we examined the changing correlations between the SSRT estimated using method of constant stimuli and SSRT estimate using each of the staircase methods, as participants completed the 40 stop trials with each staircase. Rather than simply subtracting each of the critical SSD estimates from the same overall go RT, we used a progressive go RT mean, which took the mean RT up to and including the current block. The correlations are shown in Fig 3D (again, correlations with the mean of the two Ψ methods is also shown). All estimates had correlations above 0.86 after 20 stop trials and above 0.88 after 30 trials.

### Discussion

The estimates of critical SSD and SSRT were very similar across estimation methods. The very similar results from the two Ψ estimates (using two different sigmoid functions) and the high correlations between each of these and the results from the CS method indicate that the new method produces reliable estimates of SSRT. Given that this is achieved in less than 30 stop trials, many fewer trials than either the CS or the stepwise adjustment method, the Ψ method is likely to be valuable when assessing populations with limited attention span.

While similar values were obtained across all methods, the Stepwise-adjustment method yielded a critical SSD that was significantly lower than the other methods, which corresponds to a relatively high estimate of SSRT. We suspect this was due mainly to the starting point for SSD on this staircase (60 ms) being considerably shorter than the average critical SSD estimate, biasing the average SSD to be slightly short overall, which would be consistent with the simulation results reported previously. Importantly, the estimates from this staircase still correlated highly with the CS and Ψ estimates, suggesting that all three were capturing individual variation in largely the same way, even if the stepwise SSD estimate was generally slightly lower.

Simulation results (Fig 1C & 1D) indicated that whereas the Ψ method reached an unbiased estimate of SSRT in comparatively few stop trials (< 30), the staircase method required substantially more trials, showing improvement even after 40 stop trials. The speed with which the Ψ staircase reaches a reasonably accurate and unbiased estimate is probably its biggest advantage over other methods. The method of CS is preferred over the method of adjustment when there are relatively relaxed constraints on the number of trials that can be delivered (though this is rarely the case when assessing response inhibition). Given this preference, and that the CS method was originally used in developing the SST [1], the closer match of results from the Ψ method to the CS method gives some confidence in it as an alternative to both the CS and the Stepwise-adjustment methods.

## Experiment 2

A major potential benefit of using a rapid estimator of SSD is that the stop signal task might be more effectively used to gauge inhibitory control in special populations, particularly those with diminished patience or concentration such as is found in ADHD and with young children. Towards this goal, we also examined the reliability of the Ψ staircase in using a gross motor task designed for children. The task has been used previously with a conventional staircase procedure [11, 12, 13]. Here, we applied a similar design as that used in Experiment 1, with intermixed stop trials manipulating SSD via method of constant stimuli, one staircase using stepwise adjustment, and one staircase using the Ψ method.

### Method

#### Participants and apparatus

Fifty-six undergraduate psychology students from the University of Sydney participated in the experiment, each tested individually in a quiet room. Six participants were excluded from the analyses for providing data that were poorly fit by the method of constant stimuli (using the same criterion as Experiment 1). This left a sample of 50 (30 female) of mean age 25.9 years (SD = 9.97, range: 18–59 years). Participants were tested in small groups of up to four at a time. The experiment was run on Dell Optiplex computers attached to 17-inch touch sensitive screens, using software designed using Visual Basic. Audio stimuli were again delivered through headphones.

#### Procedure

The procedure was a participant-controlled version of the task used by Pasalich et al. [13]. The go task was a matching-to-sample task in which participants had to respond to a target stimulus (a blue square or blue circle appearing in the top-center of the screen) by touching the matching shape in the top-left (blue square) or top-right (blue circle) of the screen. To initiate a trial, participants pressed the space bar, at which point a cartoon character appeared at the bottom-center of the screen. Participants had to touch the cartoon character, at which point the target stimulus immediately appeared. Participants were told to respond as fast as possible without sacrificing accuracy. Response time and errors in touching the matching shape were then recorded. If they touched the matching shape within a fixed one-second period, correct feedback was immediately presented (a smiling cartoon face and chime delivered through headphones). If the non-matching shape was touched, or no response was made within one second then incorrect feedback was presented (a frowning cartoon face and buzzer noise). On 25% of trials, a stop signal was delivered. This consisted of the same loud noise used in Experiment 1 accompanied by the target and match shapes turning red. On these trials, any touch response was met with incorrect feedback, whereas a successful timeout was met with correct feedback.

The task consisted of 12 blocks of trials, with the initial two blocks being short practice runs, introducing the go task in block 1 followed by the stop task in block 2 (as in Experiment 1). The experimental trials were then organized into 10 equal blocks, each comprising 54 go trials, 10 stop trials controlled by method of CS, 4 stop trials controlled by stepwise adjustment (50 msec steps) and 4 stop trials controlled by the Ψ adjustment method. To motivate participants to respond quickly to the target stimulus throughout the experiment, at the end of each block they were shown their mean correct response time for the last block and for several previous blocks and were told to try to maintain their speed throughout the experiment.

### Results

#### Estimates of the critical Stop Signal Delay

**Fig 4:**
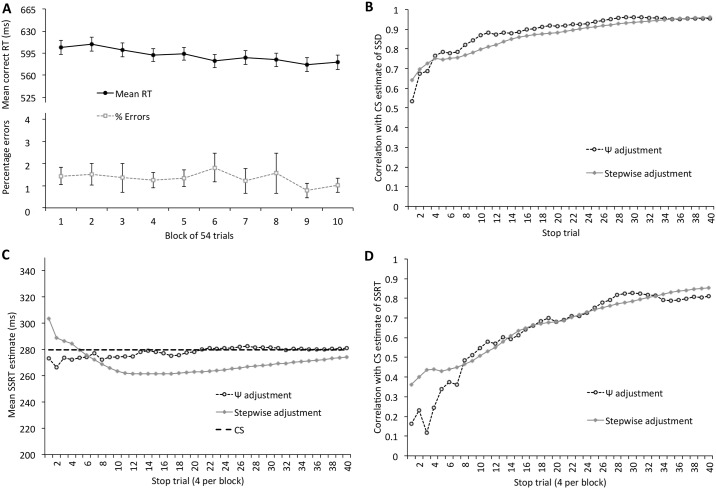
Estimates of critical SSD and response speed in Experiment 2. (A) Mean RT on correct go trials and % errors across the 10 blocks of trials. (B) Correlations between the CS estimate of SSD and the two staircases across trials. (C) Mean SSRT estimates with increasing number of stop trials in the two staircases, compared to the overall estimate produced by method of constant stimuli (CS). (D) Correlations between the CS estimate of SSRT and the two staircases across trials.

The mean critical SSD calculated using the method of constant stimuli was 310.72 ms (SD = 81.89 ms). For the Ψ staircase the mean estimate after 40 trials was 309.44 ms (SD = 85.83 ms). The mean estimate after 40 trials of the stepwise-adjusted staircase was 316.32 ms (SD = 81.97 ms). Pairwise comparisons of these estimates, correcting for family-wise error rate, found no significant differences between these estimates, largest t(49) = 1.70. Panel A of Fig 4 depicts the correlation between the SSD calculated using the method of constant stimuli and each of the staircases after 1–40 trials. Both staircases had correlations with the CS estimate greater than 0.88 after 20 trials and greater than 0.93 after 30 trials.

#### Go responses and SSRT estimates

Over the ten experimental blocks, accuracy on the primary task was very high (mean = 97.26%, SD = 3.16%) and mean RT was 590.92 ms (SD = 72.82ms). As in Experiment 1, mean RT decreased slightly across the experimental blocks (one average, 3.2 ms per block of 54 go trials).

At the end of the experimental blocks, the SSRT estimates were 279.80 ms (SD = 41.19 ms) for the method of constant stimuli, 281.08 ms (SD = 41.38 ms) for the Ψ staircase and 274.20 ms (SD = 44.42 ms) for the stepwise adjusted staircase. As with the SSD estimates, we examined the changing correlations between the SSRT estimated using method of constant stimuli and SSRT estimate using each of the staircase methods, as participants completed the 40 stop trials with each staircase. The correlations are shown in Fig 4D. The correlations were somewhat lower than the SSRT estimates in Experiment 1. After 20 stop trials, both the Ψ staircase and stepwise adjusted staircase had correlations of 0.68, rising to 0.83 and 0.79 respectively after 30 trials, and 0.81 and 0.85 respectively after 40 trials.

### Discussion

The results for the ‘child-friendly’ gross motor version of the SST used in Experiment 2 closely mirror those of Experiment 1 with strong correlations between methods on estimates of SSRT and the Ψ method yielding stable (asymptotic) estimates within 20–30 stop trials. As in Experiment 1, the Ψ method and the method of CS produced estimates of critical SSD and SSRT that were more similar than for the stepwise-adjusted staircase. In Experiment 2 the stepwise-adjusted-staircase method produced a higher estimate of critical SSD and lower estimate of SSRT than the other two procedures (the opposite of Experiment 1) but these differences were not significant. This appears to support the view that the Ψ method produces a more reliable estimate of SSRT than the stepwise-adjusted method, at least over 40 stop trials.

## General Discussion

Through simulation and human experimentation we examined the feasibility of using the Ψ adaptive staircase to estimate SSRT. The Ψ staircase could be used to replace slower conventional methods for finding the critical SSD, the interval at which a participant is equally likely to inhibit or fail to inhibit on a stop trial. Simulations suggest that the procedure generates a rapid and quite accurate estimate of the critical SSD provided that the actual value lies within the hypothesis space of the staircase. Choosing a relatively wide range of parameters for the psychometric function is thus important for maximizing the reliability of the measure. Experiments with normal adult participants, that compared performance of the Ψ staircase and a stepwise adaptive staircase against a basic method of constant stimuli, found accurate and reliable measurement using the Ψ staircase using a standard choice task (Experiment 1) and gross-motor matching-to-sample task designed for testing young children (Experiment 2). The Ψ staircase SSD and SSRT estimates correlated highly with CS estimates. The correlation between Ψ and CS estimates increased rapidly with added trials such that correlations after 20–30 Ψ trials were quite comparable to correlations between stepwise staircase and CS estimates after 40 trials. This suggests that technique could be very useful in obtaining SSRT estimates quickly.

While the advantages of the Ψ method have been demonstrated previously in perceptual paradigms (15,16), our results show that the method also has application in other areas where there is an advantage in homing in quickly upon a critical behavioural estimate. The SST is a task in which the Ψ method appears to quickly and accurately estimate the critical parameter (the SSD for which RI success and failure are equally likely) that can be used to gauge speed of response inhibition. The SST employs a highly repetitive two-choice discrimination task requiring multiple trials to achieve a reliable estimate of SSRT. Any reduction in the number of trials required for this estimate will increase the likelihood of on-target performance throughout the test and hence an increase in the reliability of the estimate. This is particularly the case for populations in which there is likely to be poor attention to such unstimulating tasks.

Performance on speeded tasks is known to change substantially with experience, even across the course of a single experimental session. Automatic learning effects are well documented in simple go/no go and choice RT tasks even when the trial sequences employed on those tasks are unsystematic (for instance, the Perruchet effect [30, 31, 32, 33]). In the SST, a combination of automatic and intentional, strategic changes in responding may occur across the course of an experimental session, such as incremental slowing of go responses, or deliberate anticipation of Stop signals (e.g. [34, 35, 36], see [37] for a review). The ability to measure SSRT quickly may mitigate this problem to some extent, at least for brief single-session tests where the experimenter cannot afford to “train out” practice effects prior to critical measurement. This adaptive staircase may therefore also prove to be useful for a range of experimental questions relating to changes in response inhibition with experience or planning.

In the experiments reported here, we saw little evidence of strategic slowing of the go response over the course of the experiment. We are confident that this is due to the emphasis we placed on continuing to respond quickly coupled with self-monitoring of go RT across blocks. However, it is possible that the presence of stop trials varied by the method of constant stimuli, which periodically presents very short and very long SSDs, may have helped to keep response speed constant as well. If this were the case then there may be some concern that using the Ψ method on its own will be associated with strategic response slowing. That is, perhaps a relatively short procedure that only presents SSDs clustered around the critical SSD would lend itself to response slowing to a greater extent, thereby invalidating the estimate produced. Although the Ψ method produces SSDs that are typically less clustered than a stepwise-adjusted staircase, it is still possible that the method, when delivered as the *only* form of stop trial, could be prone to response slowing. It is therefore worth noting that our preliminary research using this method by itself has not yielded within-task response slowing.

**Fig 5:**
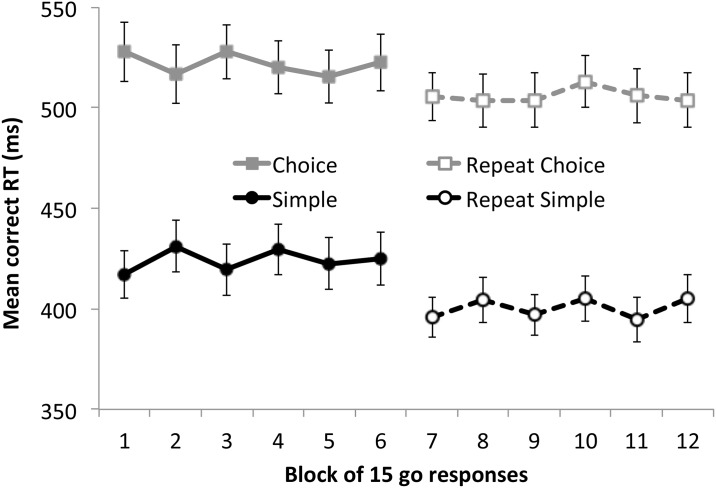
Mean go RT across blocks of test trials in two types of SST using a Ψ staircase in isolation. Both tasks were gross-motor tasks requiring touching a target stimulus on-screen. The “Simple” task involved simply touching a shape that appeared in an unpredictable location on screen (and withholding this response when the stop signal was displayed on stop trials). The “Choice” task was similar to the matching-to-sample task used in Experiment 2 of this study. Fifty undergraduate students completed both tasks in a counterbalanced order, then later repeated each task. The data are presented here merely to illustrate that it is possible to obtain relatively stable response speed while using the Ψ method in isolation (there is no evidence of strategic response slowing). Error bars show standard error.

Fig 5 shows mean correct go RT for a preliminary study in which the Ψ method was used on its own as the method for estimating the critical SSD. RT is averaged across blocks of 15 go trials in two procedures that presented 30 stop signal trials controlled by a Ψ staircase amongst 90 go trials. A full description of the study is beyond the scope of this article but to summarise briefly, the results come from 50 healthy adult participants who performed two types of gross motor task using a touchscreen. One task was very similar to the matching-to-sample task used in Experiment 2. The other was a simple gross motor task in which participants initiated each trial by touching a start button in the bottom-center of the screen and then, as quickly as possible, touched a circle appearing randomly in one of ten possible locations that were equidistant from the start button. The simple task used the same stop signal as the matching-to-sample (i.e. a loud beep accompanied by the shape changing colour). Every participant completed the two tasks, with test order counterbalanced across participants. Later in the same session, they participants repeated both tasks. The linear trends across blocks were non-significant for both tasks and for both the initial and repeat test (all *F*s < 1), suggesting that mean RTs were relatively stable across each short procedure. The repeat of each test relative to the first shows (if anything) a speeding up of RT consistent with a practice effect rather than strategic slowing. Notwithstanding these observations, the Ψ method will certainly need further validation as a standalone estimate of SSRT if it is to be relied upon as an assessment tool.

The SST has become a widely used method for measuring RI since Barkley [38] implicated inhibitory function as the primary deficit associated with ADHD. SSRT has been subsequently used as an indicator of response control, both in the study of dysfunction (as in ADHD) and in developmental studies tracing age-related changes in response control. However, the length of the conventional SSRT procedure is problematic for studying response inhibition in young children and in populations with limited executive control. The Ψ staircase could be useful in yielding estimates of SSRT with a dramatically shortened procedure. Further validation with these populations will be necessary to assess its utility.
